# A single low-dimensional neural component of spinal motor neuron activity explains force generation across repetitive isometric tasks

**DOI:** 10.1016/j.isci.2025.113483

**Published:** 2025-09-03

**Authors:** Hélio V. Cabral, J. Greig Inglis, Elmira Pourreza, Milena A. Dos Santos, Caterina Cosentino, David O’Reilly, Ioannis Delis, Francesco Negro

**Affiliations:** 1 Department of Clinical and Experimental Sciences, Università degli Studi di Brescia, Brescia, Italy; 2 School of Biomedical Sciences, University of Leeds, Leeds, West Yorkshire, UK

**Keywords:** Bioengineering, neuroscience, Systems neuroscience

## Abstract

Low-dimensional control is thought to underlie spinal motor neuron activity, with low-frequency oscillations in common synaptic inputs serving as the primary determinant of muscle force production. Here, we used principal-component analysis and factor analysis to investigate the role of low-dimensional motor unit components in force production during repetitive isometric tasks with similar force profiles. In both individual and synergistic human muscles, the first motor unit component explained most of the variance in smoothed discharge rates and showed higher correlations with force oscillations than the second component. Additionally, the first component, but not the second, remained highly consistent across trials. A non-linear network-information framework further confirmed these findings, revealing high motor unit network density in the first component across all muscles. These results suggest that during isometric contractions, force oscillations are primarily driven by a single dominant shared synaptic input to spinal motor neuron activity.

## Highlights

• We assessed the role of low-dimensional motor unit components in force production
• We explored the consistency of these components during repetitive isometric tasks
• The first component showed greater correlations with force compared to the second
• The first component, but not second, exhibited high consistency across trials

## Graphical abstract

**Figure:**
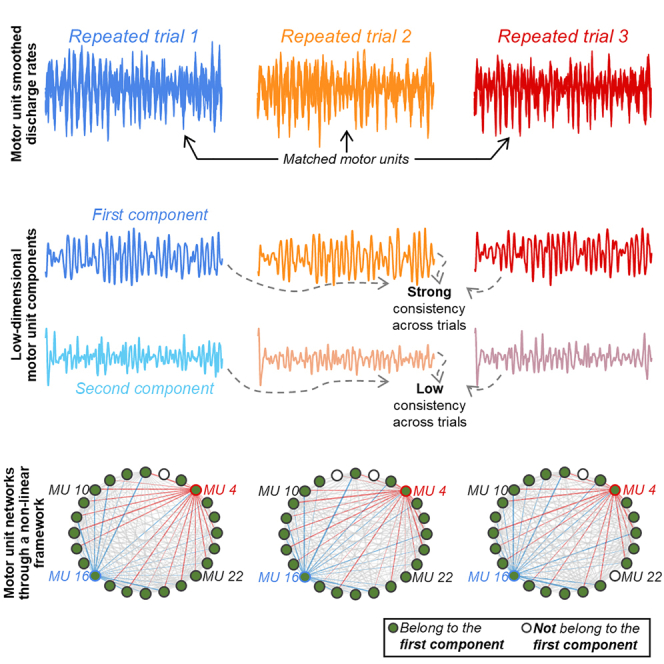

## Introduction

The central nervous system (CNS) generates and controls movement by transmitting and modulating neural commands to muscles, ensuring precise coordination and force production.^1^^,^^2^ Although movement execution often appears effortless, even simple and repetitive tasks, such as reaching or walking, involve remarkable complexity. These actions require the simultaneous activation of multiple muscles, each comprising thousands of motor units, to generate torque across multiple joints and produce purposeful motion.^3^^,^^4^^,^^5^ At every level of the motor control hierarchy, the CNS must coordinate a vast number of interacting elements while managing the hierarchical mismatch between their abundance (e.g., much greater number of alpha motor neurons than the number of muscles). While this redundancy in the neuromuscular system offers flexibility and adaptability, it also presents a complex control problem, as the CNS must navigate infinite potential solutions to achieve a given motor goal (commonly referred to as the “degrees of freedom problem”^3^).

Several theoretical frameworks have addressed the degrees of freedom problem.^6^^,^^7^ Among them, a prominent hypothesis is that the CNS employs a modular control strategy rather than individually controlling each element, reducing the complexity and dimensionality of control.^4^^,^^5^^,^^8^^,^^9^^,^^10^^,^^11^^,^^12^^,^^13^^,^^14^ One key formulation of this modular control is the muscle synergy hypothesis, which proposes that a repertoire of movements is generated by the coordinated activity of muscles, referred to as synergies, in weighted combinations.^15^^,^^16^^,^^17^ Empirical evidence supporting the existence of muscle synergies has been gathered from both animal^15^^,^^16^^,^^18^^,^^19^ and human^20^^,^^21^^,^^22^ studies, where the observed muscle activity or kinematic patterns were modeled as linear combinations of a small set of components.^23^ Despite its valuable contributions to understanding neuromuscular control, this body of research has primarily analyzed the muscles rather than the individual motor units activating them.

Recent advances in technology and analytical methods have shifted the scale of analysis from muscles to individual neurons. These developments have enabled the recording and analysis of the activity of several motor neurons at both cortical^24^^,^^25^^,^^26^^,^^27^ and spinal^28^^,^^29^^,^^30^^,^^31^^,^^32^ levels, significantly advancing the understanding of the dimensionality of neural control. At the cortical level, compelling evidence has shown that movement planning and execution are governed by a small set of neural activity patterns spanning a low-dimensional space, often referred to as the “neural manifold.”^33^ Corroborating earlier research that investigated the collective behavior of neurons,^34^^,^^35^ this evidence supports the idea that the population, rather than single neuron, is the fundamental unit underlying neural dynamics.^36^^,^^37^^,^^38^ A similar framework has been proposed at the spinal level, where examining the pool of motor units offers deeper insights into muscle force control.^39^ Simulation and experimental studies have extensively demonstrated that the alpha motor neuron pool acts as a highly selective filter, linearly transmitting the common synaptic inputs while attenuating independent inputs.^40^^,^^41^^,^^42^^,^^43^^,^^44^ These findings suggest that common synaptic inputs are the primary determinant of generated muscle force during isometric contractions, supporting the hypothesis that low-dimensional neural control extends to motor unit activity.^40^^,^^45^^,^^46^^,^^47^^,^^48^^,^^49^^,^^50^

A defining feature of low-dimensional neural control is the coupling among elements within the system, which results in a high degree of correlation among neural outputs.^51^^,^^52^^,^^53^^,^^54^ Consequently, dimensionality reduction techniques, such as principal-component analysis (PCA) and factor analysis (FA), have been widely applied to discharge rates of neuronal ensembles to identify dominant patterns of covariation, yielding a reduced set of explanatory components that capture key control features.^25^ For instance, Negro et al. (2009) demonstrated that the first principal component, derived using PCA from the smoothed discharge rates of active motor units, explains oscillations in isometric force more effectively than the discharge rates of individual motor units. In general, there exists a strong justification for using PCA to investigate patterns of neuronal correlation^40^^,^^49^^,^^55^^,^^56^ since PCA is mathematically linked to Hebbian theory,^57^^,^^58^^,^^59^ one of the most experimentally validated theories of synaptic plasticity.^35^ Specifically, Oja’s learning rule (constrained Hebbian theory) demonstrated that, over time, the synaptic weights between neurons converge to the first principal component of the high-dimensional inputs.^60^ More recent studies using alternative rotations of low-dimensional components, such as oblique rotations (e.g., Promax), or other dimensionality reduction techniques (e.g., non-negative matrix factorization) have suggested that multiple components may underlie motor neuron activity, particularly when synergistic muscles are involved.^47^^,^^50^^,^^61^^,^^62^ Interestingly, some of these recent evidence have shown that although multiple low-dimensional components can be identified in the vasti motor units, individuals were not able to volitionally dissociate motor unit activity during online control tasks.^50^^,^^62^ These findings suggest that the presence of a multidimensional motor unit manifold from dimensionality reduction does not necessarily reflect distinct volitional control signals, and, therefore, components beyond the first principal component might reflect residual variance or inputs from other spinal circuitry (e.g., recurrent inhibition^62^), which are not directly linked to force production. However, it remains uncertain whether these additional components contribute meaningfully to force production or simply reflect variance unrelated to volitional output, particularly for synergistic muscles such as the vasti. Moreover, to our knowledge, no prior study has assessed the consistency of these components across repeated tasks with highly similar motor outputs, which is an essential consideration if such components are to be interpreted as stable neural strategies to force control. Finally, most existing approaches rely predominantly on linear dimensionality reduction techniques, which impose assumptions that may not fully capture the non-linear dependencies among motor units or the inherent non-linearities in force generation.

In this study, we addressed these gaps by investigating the functional role and trial-to-trial consistency of low-dimensional components underlying motor unit activity during repetitive isometric tasks with highly similar force outputs. Specifically, we aimed to determine whether components beyond the first contribute meaningfully to force control and whether these components represent stable neural strategies by assessing their consistency across repeated trials. To characterize potential non-linear interactions among motor unit activity, we employed a recently developed non-linear framework combining information and network theoretic tools to decompose neural signals and characterize their network structure.^63^^,^^64^ We investigated these questions across different muscles, including both individual (tibialis anterior [TA], first dorsal interosseous [FDI]) and synergistic (vastus medialis [VM], vastus lateralis [VL]) muscles. VM and VL were included to examine whether similar results would emerge under shared control strategies, compared to individual muscles primarily producing force alone on a single joint. This synergistic group is particularly relevant, as VM and VL are known to receive largely shared synaptic input during isometric knee extension.^45^^,^^50^ Participants performed 15 trials of a force-matching task, following a target trajectory containing random oscillations with frequency content below 1.5 Hz (learning task). The first three consecutive trials with the lowest root-mean-square error (RMSE) between the force and target signals (i.e., post-skill acquisition trials) were selected for analysis, ensuring similarity in force oscillations across trials. Motor units were decomposed from high-density surface electromyograms (HDsEMG), tracked across the selected trials, and their smoothed discharge rates were linearly decomposed into low-dimensional components using PCA and FA. Our findings revealed that the first low-dimensional motor unit component remained highly consistent across trials and closely resembled force oscillations for all the muscles investigated, suggesting that a single dominant shared synaptic input to spinal motor neuron activity primarily controls force output.

## Results

### Similarity in force oscillations across selected trials

**Figure 1:**
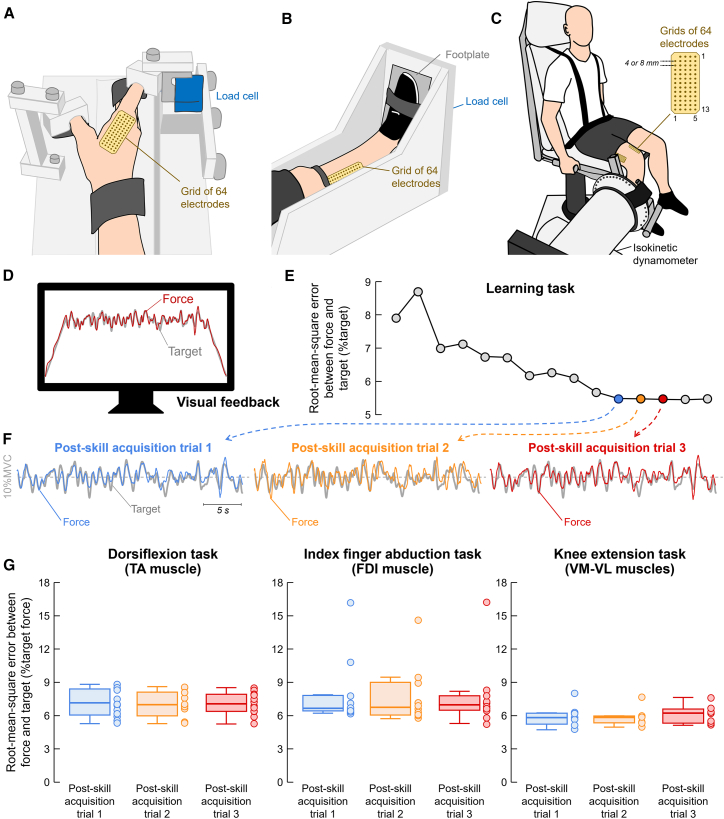
Experimental setup and similarity in force oscillations across selected trials Experimental setup to record isometric forces produced by index finger abduction (A), dorsiflexion (B), and knee extension (C) tasks. Participants were asked to perform 15 trials of a force-matching task, following a target trajectory (gray line) displayed on a computer monitor (D). From these 15 trials, the first three consecutive trials with the lowest force-target root-mean-square errors were selected for analysis (E). Comparison between force oscillations (colored lines) and the target (gray line) for the three selected trials (F). A high degree of similarity in force fluctuations across trials is evident. Group results of the root-mean-square error between the force and target are shown for the dorsiflexion (*n* = 12; Friedman test; *p* = 0.097; left panel in G), index finger abduction (*n* = 10; Friedman test; *p* = 0.741; middle panel in G), and knee extension (*n* = 7; Friedman test; *p* = 0.651; right panel in G) tasks. Circles represent individual participants. Data are represented as median (horizontal lines), interquartile range (boxes), and distribution range (whiskers). VM, vastus medialis; VL, vastus lateralis.

Figure 1 shows the experimental setups used to record isometric forces produced by index finger abduction (FDI muscle; Figure 1A), dorsiflexion (TA muscle; Figure 1B), and knee extension (VM and VL muscles; Figure 1C). During the force-matching learning task, participants followed a target trajectory displayed on a screen (Figure 1D) across 15 trials. Target force levels were set at 5% of the maximal voluntary contraction (MVC) for the index finger abduction task and 10% MVC for the dorsiflexion and knee extension tasks. The corresponding MVC torque values were as follows: index finger abduction: 1.92 ± 0.79 Nm, dorsiflexion: 40.83 ± 12.75 Nm, and knee extension: 388.83 ± 98.82 Nm. From the 15 trials, we selected the first three consecutive trials with the lowest force-target RMSE for analysis (i.e., post-skill acquisition trials; Figure 1E). On average, the first trial of the selected three occurred at trial 12 ± 1 for the index finger abduction, 11 ± 2 for the dorsiflexion, and 11 ± 2 for the knee extension.

Figure 1F provides a representative example of the force oscillations observed in the selected trials. Visually, there is a clear overlap between the force (colored traces) and target (gray traces) signals, along with a high degree of similarity in force oscillations across trials. Consistent with these observations, no significant differences in force-target RMSE were observed across trials for any of the tasks investigated (Friedman test; *p* > 0.096 for all cases; Figure 1G). Note that one participant exhibited outlier RMSE values in the index finger abduction task (Figure 1G, middle panel; values >12). When this participant was excluded from the analysis, results remained consistent, with no significant differences in RMSE across trials (*p* = 0.641). Similarly, no significant differences were observed in the coefficient of variation of force across trials (Friedman test; *p* > 0.155 for all cases). For the isometric dorsiflexion task (TA muscle), the average coefficient of variation of force values were 9.47% ± 0.42%, 9.74% ± 0.94%, and 9.73% ± 0.73% for post-skill acquisition trials 1, 2, and 3, respectively. For the isometric index finger abduction task (FDI muscle), they were 9.57% ± 2.28%, 9.80% ± 2.99%, and 9.30% ± 2.36% for post-skill acquisition trials 1, 2, and 3, respectively. For the isometric knee extension task (VM-VL muscles), the values were 8.65% ± 0.31%, 8.95% ± 0.27%, and 9.14% ± 0.61% for post-skill acquisition trials 1, 2, and 3, respectively.

### Characterization of low-dimensional motor unit control using PCA and FA

**Figure 2:**
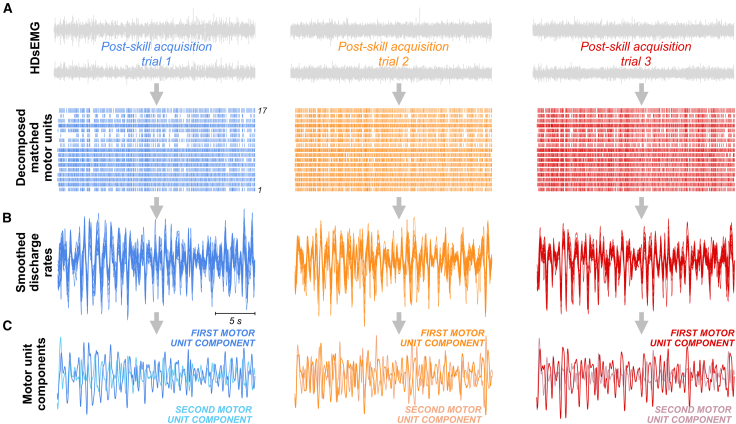
Characterization of low-dimensional motor unit control For the three post-skill acquisition trials selected for analysis, high-density surface electromyograms (HDsEMG) were decomposed into motor unit discharge times using a convolutive blind source separation algorithm (A). The motor units were tracked across trials, and their discharge times were used to calculate binary motor unit spike trains. Low-pass filtered discharge rates of tracked motor units were obtained by convolving the motor unit spike trains with a 400-ms Hanning window (B). The standardized and detrended smoothed discharge rates were then used to estimate low-dimensional motor unit components via principal-component analysis (PCA) and factor analysis (FA). Two components were extracted for all muscles analyzed (C).

To evaluate low-dimensional neural control underlying motor unit activity during repeated trials with similar force oscillations, we decomposed HDsEMG signals into individual motor unit spike trains^30^ and tracked the motor units across trials by reapplying the motor unit separation vectors^65^^,^^66^ (Figure 2A). The average number of matched motor units per participant was 16 ± 7 for the TA, 7 ± 1 for the FDI, 3 ± 1 for the VM, and 12 ± 5 for the VL. All subsequent analyses were applied to matched motor units from individual muscles (TA, FDI, and VL) as well as synergistic muscles (combined VM-VL). We then calculated the smoothed discharge rates of matched motor units by convolving the binary spike trains with a 400-ms Hanning window (Figure 2B). This operation acts as a low-pass filter with a cutoff frequency of approximately 1.8 Hz,^40^ effectively isolating the low-frequency oscillations of motor unit activity. Therefore, our analysis specifically targeted the frequency band most relevant to force generation and control.^40^^,^^41^^,^^67^ Subsequently, we assessed whether the smoothed discharge rates were suitable for factor analysis. The Kaiser-Meyer-Olkin (KMO) average values were 0.96 ± 0.03 for the TA, 0.90 ± 0.03 for the FDI, 0.87 ± 0.09 for the VL, and 0.91 ± 0.06 for the VM-VL, indicating that the smoothed discharge rate matrices for all muscles were appropriate for factorization.

To determine the number of low-dimensional components to retain, we used parallel analysis.^68^ This method indicated that the average number of components to be extracted was 1.1 ± 0.3 for the TA motor units, 1.0 ± 0.2 for the FDI motor units, 1.6 ± 0.7 for the VL motor units, and 2.0 ± 0.7 for the combined VM-VL motor units. We opted to extract two components for all the muscles investigated using PCA and FA (Figure 2C). Since the extracted components were derived from low-pass filtered motor unit discharge rates, they reflect the low-frequency oscillations in the common synaptic inputs to the motor neuron pool.^40^^,^^50^ For all muscles, the first motor unit component explained significantly greater variance in the smoothed discharge rates compared with the second motor unit component (linear mixed-effect models [LMMs]; main effect of motor unit component; *p* < 0.001 for all muscles). These differences were independent from the trial analyzed (LMMs; interaction effect of motor unit component ∗ trial; *p* > 0.412 for all muscles). The first motor unit component accounted for an average variance of 80.7% ± 6.6% for the TA, 74.5% ± 8.4% for the FDI, 55.9% ± 9.9% for the VL, and 54.3% ± 9.7% for the VM-VL. The second component explained an average variance of 4.8% ± 2.2% for the TA, 9.6% ± 3.0% for the FDI, 13.5% ± 4.2% for the VL, and 11.7% ± 3.6% for the VM-VL.

### Correlation between low-dimensional motor unit components and force oscillations

**Figure 3:**
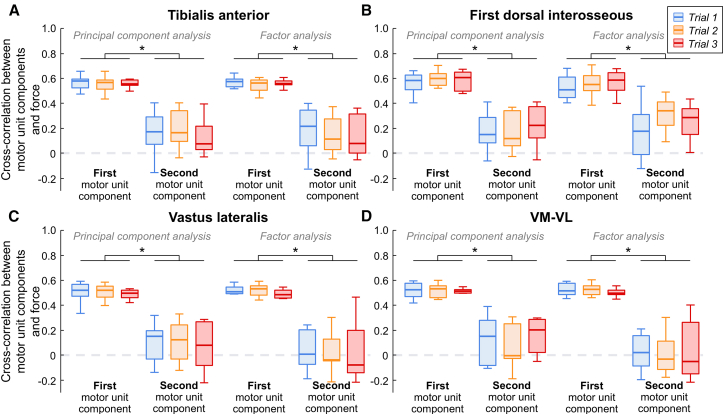
Correlation between low-dimensional motor unit components and force oscillations To evaluate how effectively the two low-dimensional motor unit components explained force oscillations, we calculated the cross-correlation between these signals. Results are presented separately by method (PCA and FA) and muscle: tibialis anterior (A), first dorsal interosseous (B), vastus lateralis (C), and combined vastus lateralis and vastus medialis (D). Data are represented as median (horizontal lines), interquartile range (boxes), and distribution range (whiskers). ∗*p* < 0.05 by linear mixed-effect models (*n* = 12 for the tibialis anterior; *n* = 10 for the first dorsal interosseous; *n* = 7 for VL and VM-VL). VM, vastus medialis; VL, vastus lateralis.

To assess how effectively the neural components underlying motor unit activity explained force oscillations, we calculated the cross-correlation between these signals. For all muscles, the first motor unit component showed significantly greater cross-correlation values with force compared to the second motor unit component (LMMs; main effect of motor unit component; *p* < 0.001 for all muscles). These differences were independent of the trial analyzed and the linear method used (LMMs; interaction effect of motor unit component ∗ trial ∗ linear method; *p* > 0.201 for all muscles). Specifically, for the TA, the cross-correlation values with force significantly increased from 0.07 (0.02, 0.13) for the second motor unit component to 0.52 (0.46, 0.57) for the first motor unit component (Figure 3A). For the FDI, the values increased from 0.22 (0.17, 0.27) to 0.56 (0.51, 0.61) between the second and first motor unit components (Figure 3B). For the VL, the increases from the second to the first motor unit component were from 0.06 (0.02, 0.11) to 0.50 (0.45, 0.54) (Figure 3C). For the VM-VL motor units, the cross-correlation increased from 0.07 (0.02, 0.13) to 0.52 (0.46, 0.57) between the second and first motor unit components (Figure 3D).

### Consistency of motor unit components across trials

**Figure 4:**
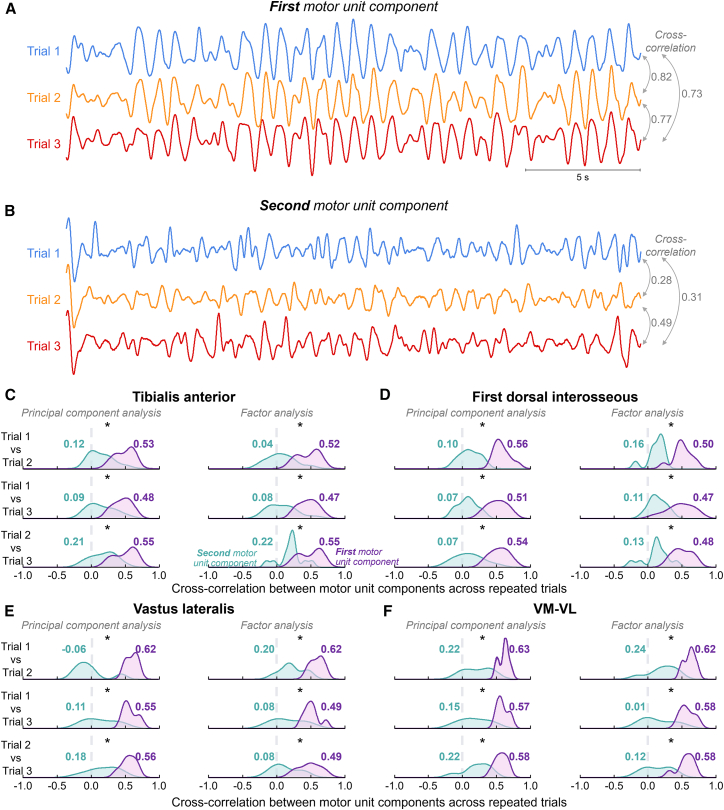
Consistency of motor unit components across trials Representative example of the first (A) and second (B) motor unit components extracted during the three post-skill acquisition trials. Note the high similarity in oscillations of the first motor unit component across trials compared with the second component. Group results of the cross-correlation are shown for the tibialis anterior (C), first dorsal interosseous (D), vastus lateralis (E), and combined vastus lateralis and vastus medialis (F). Data are represented as density curves comparing the distribution of cross-correlation values for the first (purple) and second (green) motor unit components, with average values indicated. ∗*p* < 0.05 by linear mixed-effect models (*n* = 12 for the tibialis anterior; *n* = 10 for the first dorsal interosseous; *n* = 7 for VL and VM-VL). VM, vastus medialis; VL, vastus lateralis.

To assess the consistency of the two motor unit components across trials, we computed the cross-correlation between trials. Figure 4 shows a representative case of the motor unit components extracted for each post-skill acquisition trial using PCA. While the first motor unit component (Figure 4A) remained highly consistent across trials, this similarity across trials was notably reduced for the second motor unit component (Figure 4B). This was in line with the cross-correlation values calculated between components, indicating an average of 0.78 ± 0.05 for the first component and 0.36 ± 0.11 for the second component.

Consistent with the representative case, the group results revealed significantly greater cross-correlation values across trials for the first motor unit component compared to the second component (LMMs; main effect of motor unit component; *p* < 0.001 for all muscles), regardless of the trial comparison and the linear method used (LMMs; interaction effect of motor unit component ∗ trial comparison ∗ linear method; *p* > 0.230 for all muscles). For the TA muscle, the cross-correlation values across trials significantly increased from 0.14 (0.07, 0.21) for the second motor unit component to 0.48 (0.41, 0.55) for the first motor unit component (Figure 4C). For the FDI, these values increased from 0.11 (0.06, 0.16) to 0.52 (0.46, 0.57) between the second and first components (Figure 4D). In the VL, cross-correlation across trials increased from 0.14 (0.03, 0.24) to 0.55 (0.45, 0.66) between the second and first motor unit components (Figure 4E). Similarly, for the VM-VL motor units, the cross-correlation increased from 0.18 (0.08, 0.29) to 0.58 (0.48, 0.69) between the second and first components (Figure 4F).

### Characterization of motor unit networks across trials using network-information framework

**Figure 5:**
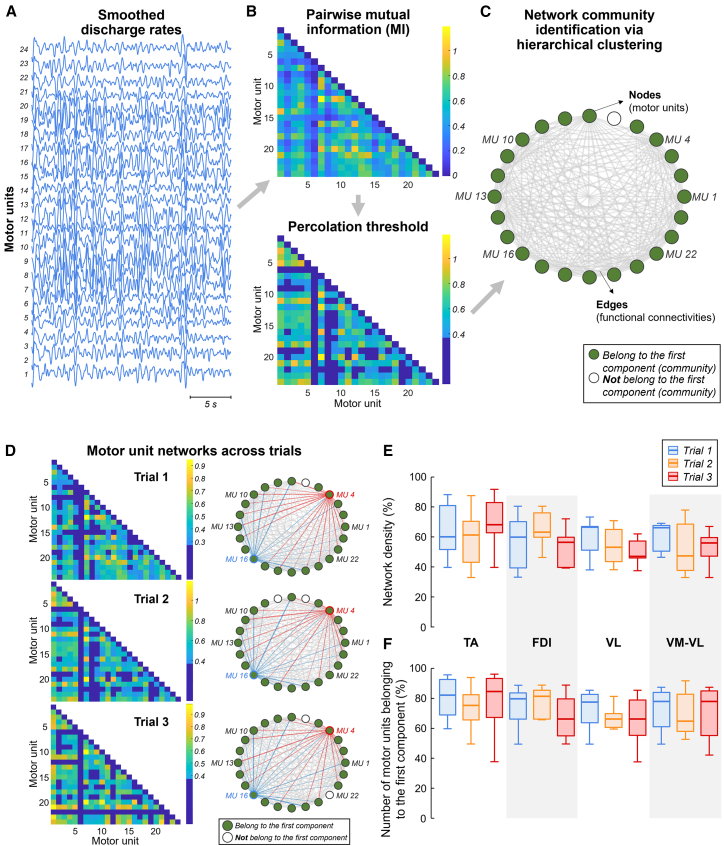
Characterization of motor unit networks across trials using network-information framework Pairwise mutual information, approximated using a Gaussian copula,^69^ was used to calculate non-linear relationships between smoothed discharge rates (A), resulting in a symmetric adjacency matrix (top panel in B). A modified percolation analysis^70^ identified significant associations between motor units (bottom panel in B). Graph theory was then applied to construct the motor unit network, where nodes (circles) represent motor units and edges (gray lines) denote significant associations (C). To identify neural components within the motor unit network, a network community detection algorithm was employed,^71^ and the percentage of motor units belonging to the first component (green nodes in C) was quantified. Representative example of non-linear pairwise mutual information analysis and the resulting motor unit networks for the three post-skill acquisition trials (D). Note the high similarity between motor unit networks, with most motor units belonging to the first component (green circles). The motor unit networks were characterized by high density (numerous connections). Group results are shown for network density (E) and the number of motor units belonging to the first component (F), separately by muscle and trial. Data are represented as median (horizontal lines), interquartile range (boxes), and distribution range (whiskers). TA, tibialis anterior; FDI, first dorsal interosseous; VM, vastus medialis; VL, vastus lateralis.

To assess the statistical dependence between pairs of motor unit smoothed discharge rates, we characterized motor unit networks across consecutive trials using a network-information framework. Briefly, the non-linear relationships between smoothed discharge rates (Figure 5A) were estimated using pairwise mutual information,^69^ resulting in a symmetric adjacency matrix representing the network-level functional connectivity between all motor units (Figure 5B). Only the significant associations between motor units were identified using a modified percolation analysis^70^ (Figure 5B), and subsequently, graph theory was employed to construct the motor unit network, where nodes represent motor units, and edges denote significant associations between motor units (Figure 5C).

Figure 5D shows the significant pairwise mutual information between matched motor units, along with the motor unit networks identified for the three consecutive trials. The networks showed a high degree of similarity across trials, as evidenced by consistent connections, such as those involving motor unit four (red edges) and motor unit sixteen (blue edges). Additionally, the motor unit networks were generally denser (i.e., featuring a greater number of connections), with most motor units belonging to the first motor unit component (or community), as indicated by the green circles representing these motor units. For the group results, the average number of identified motor unit components in the network was 1.6 ± 1.0 for the TA motor units, 1.0 ± 0.2 for the FDI motor units, 1.3 ± 0.7 for the VL motor units, and 1.4 ± 0.6 for the combined VM-VL motor units. No significant differences in network density were observed across trials for any of the muscles investigated (Friedman test; *p* > 0.145 for all cases; Figure 5E). Similarly, no significant differences were found across trials in the number of motor units belonging to the first motor unit component (Friedman test; *p* > 0.345 for all cases; Figure 5F), except for the FDI (Friedman test; *p* = 0.047). However, pairwise comparisons with Bonferroni correction did not reveal any significant differences between trials for the FDI (*p* > 0.25 for all pairwise comparisons).

## Discussion

In this study, we investigated the role of low-dimensional components underlying motor unit discharge rates in force control during repetitive isometric tasks with highly similar force outputs. First, our results showed that the first motor unit component was sufficient to explain most of the variance in smoothed discharge rates, and consistent with previous findings, it was highly correlated with force oscillations. Notably, this component also exhibited strong trial-to-trial consistency across muscles. Second, even when the second component captured a significant portion of the variance in smoothed discharge rates, it was significantly less correlated with force and showed much lower consistency across trials, thereby questioning its functional relevance in force generation. These findings were supported by a non-linear network-information framework, which demonstrated high density in motor unit networks across trials with most motor units belonging to the first component, further narrowing the functional relevancy of other components in force production. As discussed below, these results collectively suggest that, during isometric contractions, force output is primarily controlled by a single low-frequency synaptic input underlying motor unit activity.

The concept of low-dimensional neural control has been extensively studied at the muscular level, particularly through kinematics and surface electromyogram (EMG) recordings, consistently demonstrating that many movements can be explained by a small set of weighted combinations of muscle activations.^4^^,^^5^^,^^7^^,^^72^ Earlier observations of a high degree of similarity in the discharge rates of individual motor units support the idea that low-dimensional control is not confined to the muscular level but extends to the motor unit level.^52^^,^^53^^,^^73^ Initial evidence from this concept came from pairwise correlation analyses of motor unit discharge rates,^52^^,^^74^^,^^75^ which revealed that rather than acting independently, the resultant discharge times of individual motor units exhibit similar behaviors attributed to a common synaptic input, termed as common drive.^52^^,^^67^ As discussed by Miles,^76^ this dominant-shared input results from descending cortical neurons projecting broadly to the motor neuron pool and modulating a common current source injected into most or all motor neurons innervating a muscle. These insights laid the foundation for the view that common synaptic inputs across motor neuron pools are the primary source of correlated motor unit activity and, consequently, the key determinant of alpha motor neuron control.^52^^,^^73^^,^^74^^,^^75^^,^^77^^,^^78^ Subsequent simulation and experimental studies at the motor unit pool level provided further support, demonstrating that the effective neural drive to muscles is largely determined by common synaptic inputs to motor neurons.^40^^,^^41^^,^^42^^,^^43^^,^^45^^,^^50^^,^^79^ More recent research using dimensionality reduction methods has reinforced this idea, showing that one or two neural control signals are sufficient to explain the majority of motor unit activity across a range of tasks and muscles.^40^^,^^47^^,^^50^^,^^61^

Our findings align with this body of work, as we observed, using parallel analysis, that one and two components were sufficient to capture the underlying structure of motor unit discharge rates in individual (TA, FDI, and VL) and synergistic (VM-VL) muscles, respectively. Together, these components accounted for ∼85% of the variance in TA and FDI motor units, ∼70% in VL, and ∼65% in VM-VL motor units, which are values on par with previous studies.^40^^,^^47^^,^^50^ Importantly, the first component alone explained most of the variance in all muscles (∼55%–80% across trials), while the second component explained a substantially smaller portion (∼5%–10%). These results were supported by the non-linear framework, which demonstrated highly interconnected motor unit networks, with most units belonging to the first motor unit component (Figure 5). The identification of a single dominant component, through both linear and non-linear methods, suggests that the CNS employs a simplified strategy for motor unit control, where common synaptic inputs or a single dominant common drive plays a crucial role in force control. Interestingly, while a single component was retained for individual muscles, parallel analysis indicated that an average of two components was needed for synergistic control (combined VM-VL motor units). This finding aligns with other studies on the VM and VL muscles that used different methods to determine the number of low-dimensional components to retain.^47^^,^^50^^,^^61^ However, our results revealed that the second component accounted for only ∼11% of VM-VL motor unit activity, significantly less than the variance explained by the first component alone. Additionally, as further discussed below, only the first motor unit component in VM-VL largely explained oscillations in force (Figure 3D) and remained highly consistent across repeated trials with similar motor outputs (Figure 4F). Moreover, when applying non-linear methods, a single dominant component was identified on average in the motor unit networks, even for the VM-VL motor units. These results suggest that the number of extracted components for VM-VL motor units, particularly when using linear methods, does not necessarily reflect the true dimensionality of motor unit control. This finding is consistent with recent studies by Rossato et al.^50^ and Dernoncourt et al.,^62^ which showed that although multiple components were extracted from VM-VL motor units, participants were unable to dissociate motor unit pair activity during an online control paradigm. It is important to note, however, that the reduced significance of the second component compared to the first component in force control does not preclude a potential functional role. For instance, recent work by Borzelli et al.^80^ reported that more than one common synaptic input can provide distinct functional roles, such as torque production versus joint stiffness modulation, during co-contraction of antagonist muscles (biceps brachii and triceps brachii). Moreover, recent spiking network simulations suggest that the number of components identified via dimensionality reduction may reflect not only descending cortical commands but also the influence of spinal circuitry (e.g., recurrent inhibition).^62^ It is also possible that these additional components may reflect shared synaptic inputs arising from afferent feedback loop oscillations. Notwithstanding these possibilities, even if the second component in the VM-VL condition reflects a distinct functional input, our findings support the presence of a dominant common synaptic input as the main contributor to force production in the investigated muscles, which is in agreement with previous literature.^45^^,^^50^^,^^62^

Mathematically, muscle force can be modeled as the convolution of the neural drive to the muscle (i.e., the cumulative motor unit spike train) with the average twitch of active motor units.^81^^,^^82^ Although significant coherence up to 70–80 Hz has been observed in motor unit activity,^32^^,^^83^ the twitch contractile properties act as a low-pass filter on the neural drive,^84^^,^^85^^,^^86^ and, as a result, only the low-frequency oscillations in the neural drive (typically below 10–12 Hz^84^^,^^85^) are effectively transmitted to the force. Consequently, force oscillations are primarily determined by the low-frequency components of the common synaptic inputs to spinal motor neurons (for a review, see Farina and Negro^39^). From a motor unit population perspective, these low-frequency components of shared inputs can be estimated from low-pass filtered motor unit discharge rates (e.g., by convolving motor unit binary spike trains with a Hanning window), either via dimensionality reduction techniques^40^^,^^47^ or via coherence analysis between motor unit spike trains.^66^^,^^83^^,^^86^^,^^87^ Given that dimensionality reduction methods, as applied in the current study, provide time-varying signals, their fluctuations are expected to closely resemble force oscillations if they are indeed determinants of force control. Our results corroborate this hypothesis, particularly the observation that the first motor unit component was correlated with force oscillations by ∼55% across all investigated muscles (Figure 3). This correlation was consistent across trials and independent of the linear method used. Notably, only the first component closely resembled force oscillations, whereas the second component showed an average correlation of just ∼12% across muscles. These findings, consistent with previous work,^40^ strongly suggest that a single dominant common input is the primary determinant of force control and modulation, at least for the muscles investigated in this study.

An important consideration is that, despite the strong correlation between the first component and force oscillations, the correlation values did not approach 1 (i.e., perfect correlation). Several factors could explain this discrepancy. First, motor unit discharge rates are influenced by common noise inputs, which introduce variability into the neural drive signal and affect force modulation.^66^^,^^88^ Second, the muscle-tendon system introduces non-linearities that affect the translation of motor unit activity into force output.^89^^,^^90^ Third, variability in the shape of motor unit twitches may contribute to differences between predicted and actual force oscillations. In this study, smoothed discharge rates were obtained by convolving motor unit spike trains with the same Hanning window for all motor units. Previous studies have shown that using a window shape more closely resembling motor unit twitch force can yield higher correlations between the first low-dimensional motor unit component and force fluctuations.^40^ Finally, while linear methods effectively capture the patterns of shared synaptic inputs,^42^^,^^87^ they may not fully account for the non-linear dynamics inherent in force production. Non-linear methods, such as those employed in this study,^63^^,^^64^ can provide complementary insights by modeling the complex interactions within motor unit networks. For instance, the network-information framework revealed highly interconnected motor unit networks, emphasizing the role of shared inputs in driving force production (Figure 5E). Furthermore, most of the units belonged to the first motor unit component (∼80% across trials and muscles), and this pattern remained consistent across trials (Figure 5F). By capturing these higher-order interactions, non-linear methods can address limitations of linear approaches, offering a more comprehensive understanding of the neural control of force. The framework used in this study, along with high-order correlation methods,^64^^,^^91^ could be further explored in future research to provide better insights into motor unit control.

Another important finding of this study is the high consistency of the first component across trials, with average correlation values of approximately 0.6 across all investigated muscles (Figure 4). This suggests that low-dimensional control is not only effective in reducing the complexity of motor unit control but also provides a reliable and repeatable mechanism for force modulation. The high trial-to-trial consistency of the first component, but not the second, reinforces the idea that a single dominant control input driving force control is a purposeful strategy employed by the CNS to simplify coordination, particularly during repetitive tasks with similar motor outputs. There is an ongoing debate about whether low-dimensional control reflects a neural control scheme (i.e., hard-wired) or emerges as a consequence of task constraints (i.e., soft-assembled).^12^^,^^92^ Developmental studies in human neonates and infants suggest that low-dimensional patterns emerge early and may reflect innate coordination strategies.^93^^,^^94^ Additionally, recent studies in motor unit control propose that low dimensionality arises from both supraspinal and spinal mechanisms, supporting a hard-wired architecture.^62^ While our experimental approach, which focused on repetitive isometric tasks with a fixed posture, does not allow definitive conclusions regarding this question, our results indicate that the production and modulation of force after a new skill acquisition task was achieved through a single dominant control input that remained consistent across repeated trials of the same task.

An additional point worth discussing is the functional diversity of the muscles investigated. Although all four are monoarticular, they differ markedly in motor unit properties and discharge behavior.^95^^,^^96^ For example, motor units in the FDI exhibit lower innervation number^95^ and higher mean discharge rates at low force levels^96^ compared to those in the TA and vasti muscles. These physiological differences align with their functional roles: the FDI is primarily involved in fine motor control of the hand,^97^ whereas the TA and vasti are involved in gross motor functions such as posture and locomotion.^98^ This is also reflected in their maximal force capacities, with substantially lower absolute maximal forces observed during finger abduction compared to dorsiflexion and knee extension (see results). Despite these distinctions, our findings revealed remarkable consistency in low-dimensional control strategies across all muscles during the force-matching learning task. Participants typically achieved stable performance in the final third of the learning phase, with the first trial of the selected three occurring around trial 11 ± 1 for all tasks. This is consistent with recent work from our group, which demonstrated the neural mechanisms underlying short-term learning in this task are similar across hand and leg muscles.^66^ More notably, a single dominant low-dimensional component consistently explained most of the variance in motor unit discharge activity and force fluctuations across trials, regardless of the muscle’s functional role or whether it acted independently or synergistically. These results suggest that the low-dimensional neural control strategy observed here reflects a generalizable principle of force control during isometric tasks, despite differences in muscle function, output demands, and motor precision. Whether similar control strategies would apply to more complex or dynamic motor learning tasks remains an open and promising question for future research.

In this part, we would like to discuss several methodological considerations. Based on the number of trials of previous experiments involving short-term learning tasks,^66^^,^^99^ our protocol was designed to ensure consistent motor outputs across trials, which was essential for analyzing the stability of motor unit low-dimensional components. Our results indicated that performing 15 trials of the proposed task was sufficient to obtain a sequence of three post-skill acquisition trials with highly similar motor outputs (Figure 1). This was further confirmed by the absence of significant changes in force steadiness (i.e., coefficient of variation of force) between selected trials. Compared to previous studies that examined low-dimensional control of motor unit activity,^40^^,^^47^^,^^61^ our protocol provided a distinct advantage by facilitating the evaluation of the consistency of neural control strategies.

Another important methodological point concerns the determination of the number of low-dimensional components to retain.^100^^,^^101^^,^^102^ This decision is critical in dimensionality reduction, as it must balance parsimony with the ability to adequately capture the structure of the underlying data.^103^ For instance, using an arbitrary fixed threshold of explained variance (e.g., >90%) may lead to the retention of too many components, which can hinder factor interpretability and replicability.^104^^,^^105^ Furthermore, a fixed “variance accounted for threshold” has been recently shown to be insufficient to reliably estimate the dimensionality of muscle activation modules, especially in the presence of noise.^93^ Various methods have been proposed in the literature, including the “eigenvalue ≥1” criterion,^106^^,^^107^ the scree plot test,^108^ the minimum average partials criterion,^109^ parallel analysis,^110^ and consistency measures.^93^ Parallel analysis, which compares observed eigenvalues with those generated from random data, has been shown to outperform other approaches for selecting the number of components.^68^^,^^104^^,^^105^ To our knowledge, this is the first study to apply parallel analysis for selecting low-dimensional motor unit components. Given its consistency in determining the number of components across trials and muscles, as well as its data-driven nature, we recommend this approach for future studies exploring low-dimensional motor unit control.

The choice of dimensionality reduction method is another critical consideration that can affect the interpretation of results.^7^^,^^23^^,^^25^ While PCA and FA are often used interchangeably, they differ in theoretical and mathematical assumptions.^111^ PCA calculates linear combinations of measured variables (i.e., components) that maximize explained variance, including both common and unique variances. FA, on the other hand, separates common variance from unique variance, identifying latent constructs that explain shared variance among variables. Conceptually, PCA and FA differ in their approach directionality: PCA models how measured variables influence components, whereas FA assumes that latent factors drive the measured variables. For estimating commonality in motor unit discharge patterns, FA is advantageous because it focuses exclusively on shared variance across variables. However, PCA is mathematically linked to Hebbian theory,^57^^,^^58^^,^^59^ providing a solid foundation for its use in analyzing patterns of covariation within neuronal ensembles.^40^^,^^49^^,^^55^^,^^56^ Specifically, Oja’s learning rule demonstrated a biologically plausible Hebbian learning model, in which synaptic weights between two neurons adapt based on the correlation between pre- and post-synaptic activity,^35^ with a normalization constraint to prevent unbounded growth of synaptic weights.^60^ Over time, this learning rule causes the synaptic weight vector to converge to the first principal component of the input data. Thus, a simple Hebbian neuron implementing Oja’s rule effectively performs PCA on its high-dimensional inputs, supporting the use of PCA as a model of neural computation.^112^ In this study, the choice of linear method (PCA or FA) did not affect the results for any of the investigated muscles, as we focused on correlations between motor unit components and force fluctuations, as well as consistency across similar trials. Future research should select dimensionality reduction methods *a priori*, based on the specific research question and underlying assumptions.

We chose not to apply component rotations in this study, as done in recent research,^47^^,^^61^ and would like to briefly address this decision. PCA assumes that extracted components are orthogonal, whereas FA can incorporate orthogonal (e.g., varimax) or oblique (e.g., Promax) rotations to the initial factor solution. It is important to note that rotation redistributes variance across components, reducing the total variance explained by individual components due to changes in the partitioning of variance.^113^ Consequently, rotating the components affects the correlations between individual motor unit discharge rates and components (i.e., loadings). For instance, oblique rotations such as Promax may increase the correlation of certain original variables with one component while reducing it with another, thereby facilitating interpretation in specific applications where such associations may be expected (e.g., social sciences). In this study, we assumed uncorrelated components (i.e., orthogonal) for simplicity and conceptual clearness of the interpretation. In fact, if components are correlated, they may, in theory, be better interpreted as generated by a single synaptic input. Since component rotation is applied primarily to facilitate interpretation; future studies should investigate the physiological implications of perpendicular or oblique components.

Lastly, the non-linear analysis combining information theory and network-based approaches has provided novel insights into motor unit behavior. This framework has been recently applied to surface electromyograms to characterize neuromuscular networks.^64^^,^^91^ In this study, we extended this approach to motor unit discharge rates and demonstrated its potential to reveal important features of low-dimensional motor unit control during isometric contractions. By capturing complex interactions beyond traditional linear methods, this framework offers a promising tool for future research investigating motor unit network structure and its role in neural control strategies.

In conclusion, and aligned with previous research, our results provide strong evidence for low-dimensional neural control at the motor unit level, where a single dominant component is sufficient to explain the majority of motor unit discharge activity and force oscillations. Importantly, the high consistency of this component across trials further supports the idea that low-dimensional control is a reliable and efficient strategy employed by the CNS for repetitive isometric tasks. Furthermore, even when the second component captured a significant portion of variance in smoothed discharge rates, it showed lower correlation with force and poor consistency across trials, questioning its functional relevance in force generation. Future studies should investigate the generalizability of these findings to other muscles and motor tasks with greater degrees of freedom. Lastly, we offer methodological recommendations to enhance the use and replicability of linear dimensionality reduction methods in motor unit research.

### Limitations of the study

A limitation of the present study is that we only investigated the consistency of low-dimensional motor unit components during repetitive isometric tasks with similar force outputs. Future research should examine whether the same components are consistently engaged when motor units are recruited during functionally distinct tasks, such as generating forces in different directions or across varying motor goals, to better understand the flexibility of low-dimensional control strategies in force production.

## Resource availability

### Lead contact

Requests for further information and resources should be directed to and will be fulfilled by the lead contact, Prof. Francesco Negro (francesco.negro@unibs.it).

### Materials availability

This study did not generate new unique reagents.

### Data and code availability

• All individual motor unit data have been deposited at Figshare and are publicly available as of the date of publication at https://doi.org/10.6084/m9.figshare.28324253.
• This paper does not report original code.
• Any additional information required to reanalyze the data reported in this paper is available from the lead contact upon request.

## Acknowledgments

This study was funded by the European Research Council Consolidator Grant INcEPTION contract no. 101045605. J Greig Inglis was supported by the Marie Skłodowska-Curie Actions Grant “MUDecomp” agreement no. 101151712. Ioannis Delis was supported by the 10.13039/501100000268BBSRC grant no. BB/Y513799/1.

## Author contributions

Conceptualization, H.V.C. and F.N.; methodology, H.V.C. and F.N.; formal analysis, H.V.C., I.D., D.O., and F.N.; investigation, H.V.C., J.G.I., E.P., M.A.S., and C.C.; writing—original draft, H.V.C. and F.N.; writing—review & editing, H.V.C., J.G.I., I.D., D.O., and F.N.; supervision, F.N.; funding acquisition, F.N.

## Declaration of interests

The authors declare no competing interests.

## STAR★Methods

### Key resources table

**Table:** 

REAGENT or RESOURCE	SOURCE	IDENTIFIER
**Deposited data**		

All individual data of motor unit discharge times	This paper	https://doi.org/10.6084/m9.figshare.28324253

**Software and algorithms**		

MATLAB 2022b	MathWorks	www.mathworks.com
R version 4.3	R Core Team	www.r-project.org/
RStudio version 2023.06.0	RStudio Team	www.rstudio.com

### Experimental model and study participant details

#### Participants

Twenty-nine healthy participants performed a series of isometric force-matching tasks involving index finger abduction (FDI muscle), dorsiflexion (TA muscle), and knee extension (VM-VL muscles). Specifically, ten volunteers (4 females; age 31 ± 4 years; height 177 ± 8 cm; mass 72 ± 17 kg) participated in the FDI experiments, twelve volunteers (5 females; mean ± SD: age 31 ± 3 years; height 175 ± 9 cm; mass 69 ± 18 kg) in the TA experiment, and seven male volunteers (age 33 ± 10 years; height 184 ± 5 cm; mass 85 ± 19 kg) in the VM-VL experiment. Seven volunteers participated in both the TA and FDI experiments in separate sessions. All participants had no history of upper or lower limb injuries that could impact their ability to perform voluntary contractions. Before beginning the experiments, participants provided informed consent following an explanation of the experimental procedures. This study was approved by the local ethics committee of the University of Brescia (code NP5665) and conducted in accordance with the latest version of the Declaration of Helsinki.

#### Experimental protocol

For the index finger abduction task, participants were seated with their right wrist neutrally positioned on a custom-built device with their elbow flexed at 45^o^ (0^o^ being fully extended). The index finger was secured to an adjustable support attached to a load cell (SM-100 N, Interface, Arizona, USA) to record the isometric abduction force produced by the index finger (Figure 1A). To minimize the involvement of other muscles, the wrist was secured to the device with Velcro straps, and the other fingers (little, middle and ring) were strapped separately from the index finger. The thumb was fixed to an adjustable support, maintaining an approximate angle of 80° to the index finger. For the dorsiflexion task, participants were seated with their right leg positioned on a custom-made ankle dynamometer. The knee was fully extended, the hip flexed at 70° (0° being fully extended), and the ankle joint at 10° of plantar flexion (0° being the foot perpendicular to the shank). The foot was secured with straps to a footplate connected to a load cell (SM-500 N, Interface, Arizona, USA) to measure the isometric dorsiflexion force (Figure 1B). To ensure that the force generated was exclusively due to the dorsiflexor muscles, additional straps were applied around the thigh and knee. For the knee extension task, participants were seated on an isokinetic dynamometer (Humac Norm Extremity System, CSMi Solutions, Massachusetts, USA) with their right knee flexed at 90° (0° being fully extended) and aligned as coaxially as possible with the dynamometer’s axis of rotation (Figure 1C). The hip was flexed at 90° (0° being fully extended), and the ankle joint was secured to the device approximately three centimeters above the malleolus, allowing for the measurement of isometric knee extension force. The trunk and waist were also secured to the device with Velcro straps.

The experimental procedures were the same for the three muscle groups (FDI, TA, and VM-VL). Initially, participants performed a standardized warm up, consisting of three 3-s isometric contractions at 60, 70, and 80% and 90% of their subjective maximal isometric contraction, with 2 min of rest in between (adapted from Rossato et al.^50^). Then, participants performed three isometric maximal voluntary contractions (MVCs) for 3 s, with 2 min of rest between each contraction. The highest MVC among the three trials was used as a reference for the subsequent submaximal tasks. After a 5-min rest interval, participants were asked to perform an isometric force-matching task, following a complex trajectory for fifteen trials (learning task). This number of trials was chosen based on previous studies, which have demonstrated that fifteen trials of a similar task are sufficient for short-term learning.^66^^,^^99^ The trajectory specifically involved a linear increase from 0% MVC to the target force level at a rate of 5% MVC/s, a stochastic force region at the target force level for 30 s, and a linear decrease from the target force level to 0% MVC at a rate of −5% MVC/s.^66^ The stochastic force region consisted of a randomly generated signal, low-pass filtered at 1.5 Hz, with oscillations around the target force level, set at 5% MVC for the FDI muscle and 10% MVC for the TA and VM-VL muscles. The target force levels of 5% MVC (FDI) and 10% MVC (TA and VM-VL) were selected based on piloting data to minimize fatigue across the fifteen-task repetitions required for the experimental protocol. Additionally, these submaximal force levels have been commonly used in studies of low-dimensionality motor unit control, particularly in hand^40^ and lower limb muscles,^45^^,^^47^^,^^50^ facilitating comparison with previous work. Three different trajectories were generated, one for each muscle group, but the same trajectories were used for all trials and participants. A rest period of at least 1 min was provided between trials. Throughout the task, participants were encouraged by the same researcher to match the trajectory as closely as possible. Visual feedback of both the target and the produced force was displayed on a computer monitor (Lenovo ThinkVision E24-29; active area: 52.7 × 29.65 cm; 1920 × 1080 resolution), positioned at eye level approximately 1 m in front of the participant (Figure 1D). The entire target trajectory was visible from the beginning of each trial.^99^ The y axis display range was fixed from −1% to 15% MVC for the lower limb tasks and −1%–10% MVC for index finger abduction, corresponding to an amplitude resolution of approximately 0.54% MVC/cm and 0.37% MVC/cm, respectively. The time resolution (x axis) was approximately 0.61 s/cm for index finger abduction and 0.65 s/cm for the lower limb tasks. The resulting visual gain for all tasks, estimated as the visual angle subtended by the force trajectory on the screen,^114^ was approximately 0.11^o^. All participants had normal or corrected-to-normal vision and confirmed that they could clearly see both the target and the force trajectories on the screen.

#### Data collection

Monopolar high-density surface electromyograms (HDsEMG) were collected from the TA, FDI, and VM-VL muscles during the submaximal isometric tasks using adhesive grids of 64 electrodes arranged into 13 rows by 5 columns, with a missing electrode in the upper left corner (OT Bioelettronica, Turin, Italy). For the TA and VM-VL muscles, grids with an 8 mm inter-electrode distance were used (GR08MM1305), while for the FDI muscle, grids with a 4 mm inter-electrode distance (GR04MM1305) were employed. The grids were positioned longitudinally along the muscle belly, which was identified through palpation by an experienced investigator. In the case of the VL muscles, two grids were used: one positioned distally and the other proximally. Before placing the electrodes, the skin was shaved and cleaned with abrasive paste (EVERI, Spes Medica, Genova, Italy) and water to enhance signal quality. Conductive paste (AC cream, Spes Medica, Genova, Italy) was applied in the foam cavities of the grid to ensure optimal electrode-skin contact. The reference electrode was positioned on the right ankle for TA and VM-VL muscles and on the right wrist for the FDI muscle. HDsEMG and force signals were sampled synchronously at 2048 Hz using a 16-bit A/D amplifier (10–500 Hz bandwidth; Quattrocento, OT Bioelettronica, Turin, Italy).

### Method details

All analyses were conducted offline using custom-written scripts in MATLAB (version 2022b; The MathWorks Inc., Natick, Massachusetts, USA).

#### Trial selection for analysis

Three consecutive trials were selected out of the fifteen performed, focusing on those after learning the force-matching skill (i.e., post-skill acquisition trials). Initially, the force signal was low-pass filtered at 15 Hz using a third-order Butterworth filter. The RMSE between the detrended force and target signals was then calculated considering the 30-s middle region of each trial. The first three consecutive trials with the lowest RMSE between the force and target signals were selected for further analysis, as the force oscillations are expected to be most similar across these trials (Figure 1E). For these selected trials, the coefficient of variation of force (i.e., standard deviation divided by mean) was calculated to quantify force steadiness.

#### HDsEMG decomposition and motor unit tracking

To ensure the inclusion of motor units with stable discharge behaviors during the task, we excluded the first 5 s of the 30-s middle region. This step eliminated motor units that were recruited shortly before or near the target force level and may not have tonically discharged across the task. Thus, only motor units consistently active across the last 25-s of the middle region were included for analysis. Initially, monopolar HDsEMG signals were filtered between 20 and 500 Hz using a third-order bandpass Butterworth filter (Figure 2A). All signals were then visually inspected, and those of low quality (e.g., artifacts or skin-electrode problems during acquisition) were discarded from further analysis. The remaining HDsEMG signals were decomposed into motor unit discharge times using a convolutive blind-source separation algorithm,^30^ which has been previously validated and widely applied to assess individual motor units in the muscles investigated in this study.^30^^,^^66^^,^^115^ The identified motor units were visually inspected by an experienced operator, and missing or misidentified discharges (inter-spike intervals <20 ms or >250 ms; Negro et al.^40^) were manually and iteratively edited.^115^^,^^116^ Motor units were then tracked across the three trials by reapplying the motor unit separation vectors, following procedures used in previous studies.^65^^,^^66^^,^^117^ Briefly, the deconvolution of HDsEMG signals using independent component analysis involves the calculation of a separation matrix, whose columns are the separation vectors for each motor unit.^30^^,^^118^ These separation vectors are unique for each motor unit and define the spatiotemporal filters that, when applied to the HDsEMG signals, yield the estimated motor unit discharge times. To maximize the number of tracked motor units, the estimated separation vectors from one trial were applied in the other two trials, considering all possible combinations. However, in a few cases (1 participant for TA, 3 for FDI and 2 for the VM), it was not possible to track more than one unit using this method. In such cases, motor units were tracked based on their action potential shapes.^115^ Specifically, the two-dimensional representation of the motor unit action potentials identified in one trial was estimated using the spike-triggered averaging technique and then cross-correlated with the spatial representation of the motor units identified in the other two trials. Motor units with high similarity between action potentials (cross-correlation >0.8^115^) were considered to belong to the same motor unit.

#### Smoothed discharge rate matrices

The discharge times of the motor units tracked across the three post-skill acquisition trials were used to compute binary motor unit spike trains, where a value of 1 indicates the presence of a motor unit discharge at a specific time point, and a value of 0 indicates its absence (Figure 2A). The low-pass filtered discharge rates of motor units were then calculated by convolving the binary motor unit spike trains with a 400-ms Hanning window.^40^ To remove offsets and trends, these smoothed discharge rates were high-pass filtered at 0.75 Hz using a third-order Butterworth filter^52^ and subsequently standardized to have mean 0 and standard deviation 1 (Joliffe and Morgan^119^; Figure 2B). The resulting standardized and detrended smoothed discharge rates, referred to as smoothed discharge rates for simplicity, were then arranged in an *r* × *c* matrix, where *r* is the number of time samples and *c* is the number of motor units. This matrix was used to estimate the neural components underlying the low-frequency oscillations of motor units by applying linear dimensionality reduction techniques, specifically PCA and FA. Additionally, a non-linear method (network-information framework) was employed to characterize motor unit networks across trials.

#### Linear dimensionality reduction techniques

Two linear dimensionality reduction techniques, PCA and FA, were used to extract the components underlying the smoothed discharge rates of motor units.^119^^,^^120^ An important step when applying PCA or FA is to determine the number of low-dimensional components to retain. Various methods have been proposed to determine this number, often based on the eigenvalues of each component.^106^^,^^107^^,^^108^^,^^109^^,^^110^ Given previous evidence suggesting that parallel analysis is one of the most accurate methods for identifying the ideal number of components, significantly outperforming other approaches,^68^^,^^104^^,^^105^ we employed this method to determine the number of motor unit components to extract. For a detailed tutorial on performing parallel analysis, refer to Hayton et al*.*^68^ Briefly, we simulated a matrix of random data by shuffling the time samples of the smoothed discharge rate matrices calculated for each trial (see calculation of smoothed discharge rate matrices). This random data matrix was then subjected to PCA, and the estimated eigenvalues for each component were stored. This procedure was repeated 1,000 times, resulting in a set of random eigenvalues from which the average and 95% confidence intervals were calculated. The number of motor unit components to retain was determined by identifying the eigenvalues extracted from the real smoothed discharge rate matrices that exceeded the upper bound of the 95% confidence interval of the random eigenvalues. This approach was performed separately for each of the three selected trials, and the average across trials was calculated and retained for further analysis.

Based on parallel analysis, which indicated extracting an average of one motor unit component for individual muscles and two components for the VM-VL muscles (see results), we extracted two components for all analyses (Figure 2C). PCA and FA were applied without rotation to motor units decomposed from individual muscles (TA, FDI, and VL) as well as synergistic muscles (combined VM-VL). The VM muscle was not analyzed separately due to the low number of motor units matched across trials (see results). To investigate the association between force oscillations and the two motor unit components, the cross-correlation between the detrended signals was calculated.^40^ To assess the consistency of the two motor unit components across trials, cross-correlation between trials was computed using 5-s non-overlapping windows, with the resulting values averaged. Additionally, the percentage of variance in the smoothed discharge rates explained by the two components obtained through PCA was calculated and retained for analysis.

#### Network-information framework

To non-linearly characterize the connectivity between motor unit smoothed discharge rates, we applied a framework combining information and network theories (i.e., the network-information framework). The detailed methodology for this approach has been described in previous studies.^63^^,^^64^ First, the non-linear relationships between smoothed discharge rates (Figure 5A) were estimated using pairwise mutual information with a Gaussian copula-based approximation,^69^ resulting in a symmetric adjacency matrix representing the connectivities between all motor units (i.e., network-level functional connectivity; Figure 5B). A modified percolation analysis^70^ was then applied to determine a threshold (the percolation threshold), identifying only the significant associations between motor units (Figure 5B). Subsequently, graph theory was employed to construct the motor unit network, where nodes (or vertices) represent motor units, and edges (or links) denote significant associations between motor units (Figure 5C). We used a circular representation to visually observe the matched motor units across trials. As in the PCA and FA, this analysis was applied to motor units decomposed from individual muscles (TA, FDI, and VL) as well as synergistic muscles (combined VM-VL).

To identify the neural components (or communities) within the motor unit network of each post-skill acquisition trial, we applied a network community detection algorithm.^71^ This algorithm quantifies overlapping components through hierarchical clustering of network links and outputs a binary matrix, where the rows represent the identified components, and the columns represent the motor units in the network. A motor unit was assigned a value of 1 if it belonged to a specific component and 0 otherwise. This process was performed separately for each trial, and the average number of identified components across the three trials was calculated. Two metrics were used to characterize the motor unit networks for each post-skill acquisition trial. First, we calculated the network density, which quantifies the number of edges in the network relative to the total possible number of edges. A higher network density indicates stronger interconnections between nodes, reflecting a more cohesive network. Additionally, we quantified the percentage of motor units belonging to the first component (represented by green nodes in Figure 5C).

### Quantification and statistical analysis

All statistical analyses were performed using R (version 4.3) within the RStudio environment (version 2023.06.0). Since force-target RMSE and coefficient of variation of force data were not normally distributed (Kolmogorov-Smirnov test; *p* < 0.002 for all), Friedman tests with Bonferroni’s correction for post hoc pairwise comparisons were used to assess differences across the three post-skill acquisition trials. Note that similar results were obtained when using one-way repeated-measures ANOVA (not reported). We also The Kaiser-Meyer-Olkin (KMO) measure of sampling adequacy was applied to assess whether the smoothed discharge rate matrices were factorable.^121^ KMO values greater than 0.70 were considered indicative of matrices appropriate for factorization.^111^

Linear mixed-effect models (LMMs) were used to compare the total variance explained in the smoothed discharge rates by the first and second motor unit components. Random intercept models were applied, with trial (trial 1, trial 2, and trial 3) and motor unit component (first and second motor unit components) as categorical fixed effects, and participant as a random effect. To compare the cross-correlation values between motor unit components and force, random intercept models were also used, with trial (trial 1, trial 2, and trial 3), motor unit component (first and second motor unit components) and linear method (PCA and FA) as categorical fixed effects, and participant as a random effect. Similarly, to compare the cross-correlation values of motor unit components across trials, random intercept LMMs were used, with trial comparison (trial 1 vs. trial 2, trial 1 vs. trial 3, and trial 2 vs. trial 3), motor unit component (first and second motor unit components) and linear method (PCA and FA) as categorical fixed effects, and participant as a random effect. For all statistical analyses, both main effects and interactions between fixed factors were tested. LMMs were implemented using the package *lmerTest*^122^ with the Kenward-Roger method to approximate the degrees of freedom and estimate the *p*-values. The *emmeans* package was used for multiple comparisons and to calculate estimated marginal means with 95% confidence intervals.^123^

To compare network density and the number of motor units belonging to the first component across trials, Friedman tests were performed with Bonferroni’s post hoc correction for pairwise comparisons. All the statistical details are reported in the results and figure legends. All individual data of motor unit discharge times recorded for each muscle in the three post-skill acquisition trials are available at *https://doi.org/10.6084/m9.figshare.28324253*.
